# Improved muscle function and quality after diet intervention with leucine-enriched whey and antioxidants in antioxidant deficient aged mice

**DOI:** 10.18632/oncotarget.7800

**Published:** 2016-02-29

**Authors:** Miriam van Dijk, Francina J. Dijk, Annelies Bunschoten, Dorien A.M. van Dartel, Klaske van Norren, Stephane Walrand, Marion Jourdan, Sjors Verlaan, Yvette Luiking

**Affiliations:** ^1^ Nutricia Research, Nutricia Advanced Medical Nutrition, Utrecht, The Netherlands; ^2^ Department of Animal Sciences, Human and Animal Physiology, Wageningen University, Wageningen, The Netherlands; ^3^ Nutrition and Pharmacology, Wageningen University, Wageningen, The Netherlands; ^4^ Unite de Nutrition Humaine, INRA-UdA, Clermont-Ferrand, France

**Keywords:** sarcopenia, skeletal muscle, fatigue, antioxidants, Gerotarget

## Abstract

Antioxidant (AOX) deficiencies are commonly observed in older adults and oxidative stress has been suggested to contribute to sarcopenia. Here we investigate if 1) low levels of dietary antioxidants had a negative impact on parameters of muscle mass, function and quality, and 2) to study if nutritional interventions with AOX and/or leucine-enriched whey protein could improve these muscle parameters in aged mice. 18-months-old mice were fed a casein-based antioxidant-deficient (lowox) diet or a casein-based control-diet (CTRL) for 7 months. During the last 3 months, lowox-mice were subjected to either: a) continued lowox, b) supplementation with vitamin A/E, Selenium and Zinc (AOX), c) substitution of casein with leucine-enriched whey protein (PROT) or d) a combination of both AOX and PROT (TOTAL). After 7 months lowox-mice displayed lower muscle strength and more muscle fatigue compared to CTRL. Compared to lowox-mice, PROT-mice showed improved muscle power, grip strength and less muscle fatigue. AOX-mice showed improved oxidative status, less muscle fatigue, improved grip strength and mitochondrial dynamics compared to lowox-mice. The TOTAL-mice showed the combined effects of both interventions compared to lowox-mice. In conclusion, nutritional intervention with AOX and/or leucine-enriched whey protein can play a role in improving muscle health in a AOX-deficient mouse model.

## INTRODUCTION

During the ageing process, muscle mass, strength and function decline, defining sarcopenia [1]. Contributors to sarcopenia are multi-factorial: decreased levels of physical activity, increased levels of oxidative stress, pro-inflammatory status, endocrine changes, anabolic resistance, and inadequate nutrition [1]. In addition to muscle mass and strength, another determining factor of muscle function is muscle power. The definition of muscle power is the ability to perform muscular work per unit of time [2]. Muscle power is strongly associated with gait speed [3], balance [4] and functional status [5]. More importantly, muscle power has been found to be more relevant than muscle strength for many tasks of daily living [5,6]. The decline of muscle mass, strength and power suggests a progressive worsening of muscle quality during ageing or in other words the decreased capacity of the muscle to adapt to its environment [2].

One of the mechanisms that contributes to the decreased muscle mass and quality during ageing is an imbalance between muscle protein synthesis and degradation [7]. Elderly people have deficits, e.g. anabolic resistance [7-11], which hinder them from responding adequately to anabolic stimuli such as insulin, leucine or dietary protein [7]. Among the amino acids, leucine can stimulate muscle protein synthesis by improving activation of translation initiation [12] [13]. Studies in isolated rat muscles [14], in adult rats [15] and older adults [16] demonstrated that the muscle protein synthesis response can be increased by increasing leucine availability to the muscle. Whey protein, a fast digested and absorbed leucine-rich protein, has proven to be more effective in stimulating muscle protein synthesis in older adults than slow digested casein protein [17, 18]. A leucine-enriched whey protein may, therefore, represent a nutritional strategy for limiting muscle protein loss during ageing.

Another underlying mechanism of impaired muscle quality in the elderly is an increase in oxidative stress [19-21]. Oxidative damage induced by oxidative stress increases during ageing [22, 23] and can induce alterations in DNA, lipids and proteins [24], resulting in a decrease in their biological function. This may contribute to reduced muscle protein synthesis and mitochondrial dysfunction. The maximal rate at which an individual consumes oxygen (VO_2max_) declines with age, even after correcting for losses in lean mass [25]. These data suggest that the muscle mitochondrial function decreases as people age, which can be explained by impaired adaptive capacity of mitochondrial functioning [26]. Processes that are suggested to underlie this impaired adaptive capacity are decreased mitochondrial biogenesis, but also decreased mitochondrial quality control *via* lowered mitochondrial dynamics [27].

Bolstering the antioxidant defense mechanisms by supplementing the diet with antioxidants showed promising results in studies in ageing rats [28, 29]. Improved motor function and muscle protein metabolism were observed when a combination of vitamins E and C [28] or vitamins A, E with selenium and zinc was given. However, supplementation of vitamin E alone failed to attenuate oxidative damage in muscle of ageing mice [30] and only slightly attenuated an exercise-induced increase in muscle oxidative stress [31, 32]. These first indications suggest that mitochondrial functioning can be improved *via* nutritional interventions. Therefore we chose to use a mixture of plural components with antioxidant properties, which may represent the best strategy to address oxidative stress. This is even more important since older people are at risk of being deficient in multiple antioxidants, i.e. vitamins A, E, C, Selenium and Zinc [33-36]. Moreover, low levels of these antioxidants have been associated with sarcopenia and disability [37-39].

From literature, it is known that aged mice display loss of muscle mass, strength and function during ageing [40, 41]. Our previous study also showed that, compared to 10 month-old mice, normal aged 25 month-old mice have decreased plasma levels of vitamin A and E in combination with elevated levels of malondialdehyde (MDA, a marker for damage of lipid oxidation), indicating elevated oxidative stress levels in aged mice [42, 43]. The aim of the present study was two-fold. First, to determine in ageing mice the long-term effects of low dietary intake of vitamin A, E, selenium and zinc, all components with antioxidant properties [28, 29, 44-46], on muscle mass, strength, function and to mimic human ageing. In this model dietary deficiency was induced by decreasing intake of vitamin A, E, Selenium and Zinc to 25% of the daily recommended intake for rodents [47]. In this way we aimed to induce in a relatively short time interval effects on muscle parameters without completely eliminating the micro-nutrients from the diet. Secondly, this study aimed to determine the subsequent effects of dietary supplementation/ repletion with these antioxidants, or a diet replacing casein protein by leucine-enriched whey protein, or a combination of both on muscle mass, strength, function and quality. We hypothesized that aged mice on a diet low in antioxidant have more pronounced loss of muscle quality than aged mice on a control feed with standard anti-oxidant levels (AIN93M). Moreover, we tested whether the dietary interventions might improve muscle quality through improved muscle protein synthesis and/or mitochondrial dynamics [48]. Therefore we adjusted the algorithm introduced by Barbat-Artigas *et al*. to be applicable to mice [2].

## RESULTS

### Effect of diet low in components with anti-oxidant properties compared to control aged mice

#### Oxidative status

Lowox-mice did not show elevated levels of whole body oxidative stress compared to control mice at 22 months of age, as shown in Figure 3A for hepatic GSH and in Figure 3B for hepatic MDA. In the ageing process control mice had significantly higher whole body oxidative stress and lipid peroxidation, as reflected by decreasing liver GSH levels (*P* < 0.05) (Figure 3A) and increased MDA levels between 22 and 25 months (*P* < 0.05) (Figure 3B). Lowox-mice and control mice showed a similar pattern during ageing for GSH levels; however, MDA levels after 7 months of the lowox-diet were significantly higher in lowox than in control mice (*P* < 0.05). Plasma levels of vitamin A and E did not alter during ageing from 22 to 25 months, nor were they affected by the diet (Table 3). Hepatic levels of vitamin A also did not show any significant changes at 25 months of age, but vitamin E displayed a trend (*P =* 0.076) towards decreased levels in the lowox group *vs*. control.

**Table 1:** Composition of the intervention diets

Diet name	CTRL	Lowox	AOX	PROT	TOTAL
**Ingredients**	*g/kg dry matter*				
**Cornstarch**	466	466	466	466	466
**Dextrinized cornstarch**	155	155	155	155	155
**Casein**	141.8	141.8	141.8	-	-
**Whey**	-	-	-	136.1	136.1
**Leucine**^1^	13	13	13	16.8	16.8
**Sucrose**	100	100	100	100	100
**Corn oil**	40	40	40	40	40
**Fiber**^2^	50	50	50	50	50
**Mineral mix**^3^	35	35	35	35	35
**Selenium (mg/kg)**	0.150	0.0375	2	0.0375	2
**Zinc (mg/kg)**	10	2.5	65	2.5	65
**Vitamin mix**^3^	10	10	10	10	10
**Vitamin A (IU/kg)**	2400	600	8000	600	8000
**Vitamin E (IU/kg)**	20	5	500	5	500
**Choline bitrate chloride**	2.3	2.3	2.3	2.3	2.3
***tert*-butylhydroquinone**	0.008	0.008	0.008	0.008	0.008

The control diet is AIN93-M without the antioxidant L-cysteine added. The control and lowox diets were provided the first 4 months of the experiment. The lowox diet was based on the control diet, except for the vitamin and mineral mixes. For the last 3 months of the experiment the lowox group was divided in 4 groups: Lowox (continuation), AOX (antioxidant supplemented group), PROT (leucine-enriched whey protein substituted group) and TOTAL (combination of AOX and PROT strategy). All diets were equal in amounts of protein (124g), carbohydrates (674g), fat (41g) and kcal (3665) per kg dry matter.

1 Total leucine amount, from intact protein and free leucine;

2 Fiber source is cellulose;

3 AIN93-M mineral and vitamin mix [45]

**Table:** Muscle quality criteria in aged mice

Level	*In vivo* max grip strength (g)/lean mass (g)	Maximal force production (N)/EDL muscle mass (g)
Normal > 1 SD	> 0.96	> 3.24
1 SD ≥ Low > 2 SD	0.96 ≥ X > 1.92	3.24 ≥ X > 6.48
Poor ≤ 2 SD	≤ 1.92	≤ 6.48

Normal levels derived from results of a previous experiment where muscle parameters of 10 months aged mice (*n* = 48) were analyzed.

**Table:** Plasma levels of vitamin A and E at t4 (22 months of age) and t7 (25 months of age)

			Plasma		Liver	
Groups	n	Age (mo)	Vitamin A (μmol/L)	Vitamin E (μmol/L)	Vitamin A (μg/g sample)	Vitamin E (μg/g sample)
Control	7	22	2.07±0.21	6.10±0.47	956±476	26.2±5.90
Lowox	7	22	1.57±0.26	6.99±0.41	480±499	9.80±1.80^*^
						
Control	7	25	1.74±0.16	6.39±0.71	2913±285	21.2±12.2
Lowox	9	25	1.92±0.13	6.12±0.32	1851±228	5.50±0.89
AOX	9	25	1.77±0.07	11.3±0.64*	7438±651*	147±51.5*
PROT	8	25	1.83±0.22	7.10±0.41	1780±446	9.86±4.18
TOTAL	8	25	2.12±0.10	14.7±0.66*	8298±362*	373±138*

* Statistical significant effect of diet aged matched control (mixed model with post hoc Sidak, *P*<0.05)

**Figure 1:**
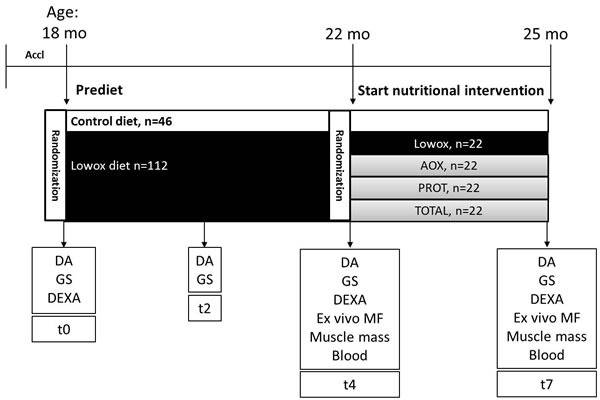
Experimental setup Abbreviations mo: months, Ctrl: mice on control diet = AIN93-M (casein protein based), Lowox: mice on diet low in antioxidants (vitamin A, E, Se and Zn), AOX: mice on diet supplemented with vitamin A, E, Se and Zn, PROT: mice on diet where casein is replaced by leucine-enriched whey protein, TOTAL: mice on diet of combination of AOX and PROT, DA: daily activity measurements, GS: *in vivo* muscle grip strength measurements, DEXA: body composition analyses, and MF: *ex vivo* muscle function measurements.

**Figure 2:**
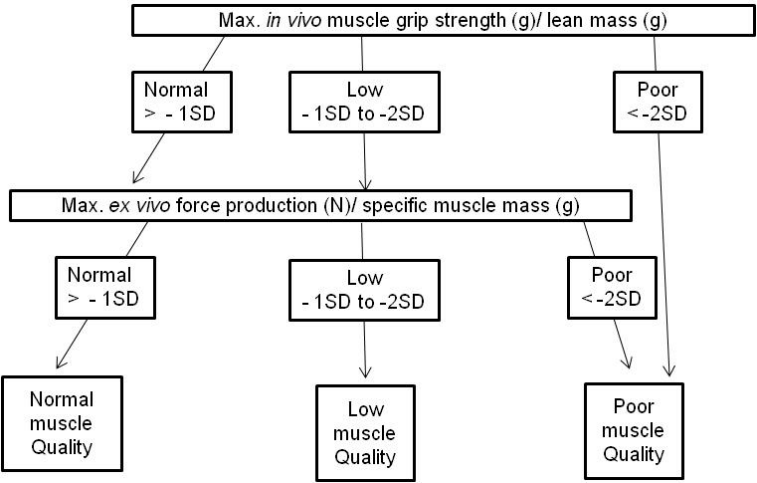
Modified algorithm to define muscle quality index in mice based on Barbat-Artigas *et al*. [2]

**Figure 3:**
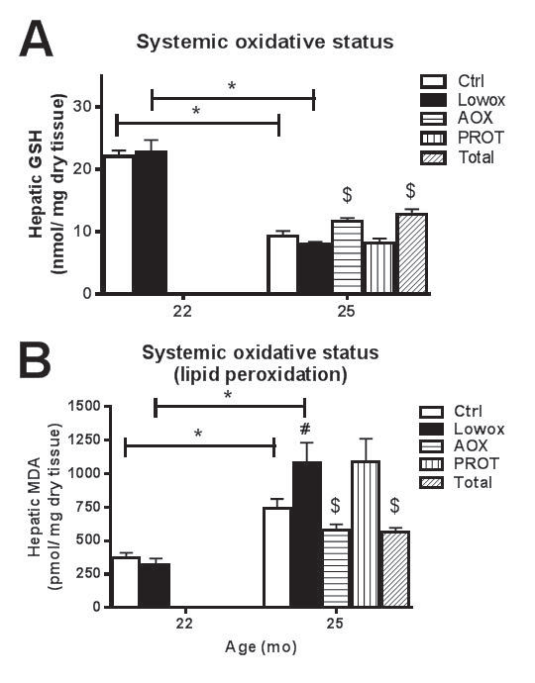
Oxidative stress **A.** hepatic total GSH levels, and **B.** hepatic MDA levels. At t0 and t4: ctrl *n* = 8 and lowox *n* = 9; at t7: ctrl *n* = 19, lowox *n* = 18, AOX *n* = 18, PROT *n* = 14 and TOTAL *n* = 17. * Statistically significant age effect, # diet effect at corresponding time point, $ nutritional intervention effect compared to lowox-diet at t7 (mixed model *post hoc* Sidak, *P* < 0.05).

#### Body weight and composition

**Figure 4:**
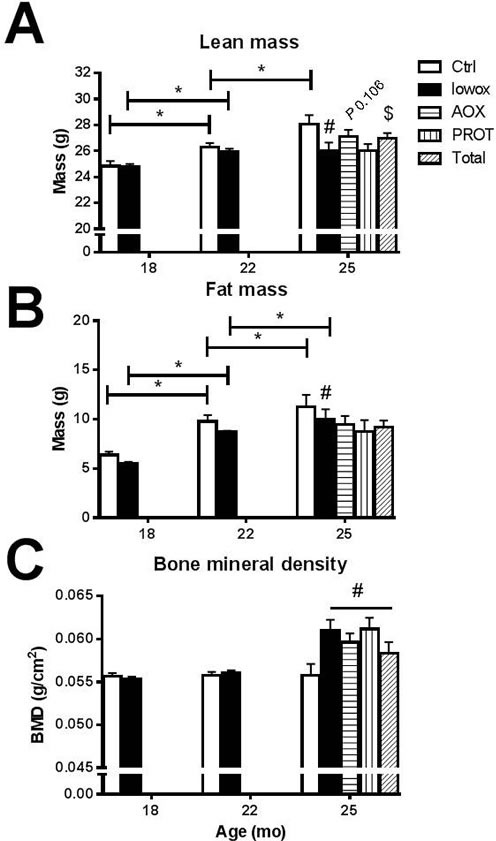
Body composition analyzed by DEXA **A.** Lean mass; **B.** fat mass and **C.** bone mineral density. At t0 and t4: ctrl *n* = 43 and lowox *n* = 42, at t7: ctrl *n* = 19, lowox *n* = 18, AOX *n* = 18, PROT *n* = 14 and TOTAL *n* = 17. * Statistically significant age effect between 2 time points, # significant effect of lowox compared to control diet at corresponding time point, $ nutritional intervention effect compared to lowox-diet at t7 (mixed model with *post hoc* Sidak, *P* < 0.05).

No significant difference in body weight was observed between control and lowox-mice, as shown in Table 4. From 18 until 22 months of age, both groups showed a significant increase in body weight (*P* < 0.0001), and thereafter, body weight was stable until 25 months of age. The control and lowox-mice did not show a statistically significant difference in food consumption during the 7 months of the experiment (Table 4). The increase in body weight during the first 4 months of the diets for both groups can be explained by a significant increase of both lean (*P* < 0.05) and fat mass (*P* < 0.0001) (Figure 4A and 4B). Although body weight was not significantly altered after 22 months of age, control mice still showed significant increases in fat and lean mass from 22 to 25 months of age. In contrast, lowox-mice did not show an increase in lean mass from 22 to 25 months of age, but fat mass increased significantly (*P* = 0.047). The lowox-mice showed a significant lower lean mass and fat mass compared to control mice at 25 months of age (*P* = 0.035 and *P* = 0.003, respectively). Surprisingly, all mice fed the lowox-diet for the first 4 months, showed a significantly higher bone mineral density compared to control mice at 25 months of age (*P* = 0.026 for overall diet effect), regardless of the nutritional intervention received for the last 3 months (Figure 4C). No significant change was observed in bone mineral content (data not shown).

**Table:** Body weight, food intake and muscle mass

Group	n	BW (g)	FI (g)	SUM (mg)	TA (mg)	EDL (mg)	SOL (mg)	PLANT (mg)	GM (mg)
Mean weights at start of experiment (pre-diet t0)									
Ctrl	41	33.9±0.5	4.07±0.04	-	-	-	-	-	-
Lowox	94	32.7±1.2	4.09±0.04	-	-	-	-	-	-
Mean weights after 4 months of lowox diet (t4)									
Ctrl	16	39.9±0.8*	4.25±0.08	481.6±13	54.3±1.1	11.8±0.3	11.5±0.5	18.6±0.6	144.7±5.0
Lowox	16	38.3±1.2*	4.13±0.08	495.2±8.0	55.1±1.3	12.3±0.3	11.9±0.4	18.9±0.4	149.5±2.9
Mean weights after additional 3 months of nutritional intervention (t7)									
Ctrl	17	39.4±2.9	4.11±0.04	482.2±10	54.4±1.2	12.6±0.3	11.5±0.4	19.1±0.7	144.3±4.1
Lowox	13	40.0±1.4	4.10±0.04	477.6±12	53.6±1.1	12.1±0.3	11.1±0.5	18.6±0.7	143.4±4.5
AOX	15	40.6±1.1	4.15±0.06	464.9±9.0	52.8±1.4	12.3±0.3	10.7±0.4	17.7±0.5	138.9±3.5
PROT	13	38.3±1.4	4.15±0.06	451.4±10	51.9±1.1	12.1±0.3	10.5±0.3	18.1±0.4	133.1±3.8
TOTAL	16	40.1±1.0	4.12±0.06	466.6±5.0	55.2±0.9	12.6±0.3	10.8±0.3	18.1±0.5	136.5±1.8

* Statistically significant age effect on BW (no diet effect) from t0 (18 months of age) to t4 (22 months of age) (repeated measurements with post hoc Sidak, P<0.05); no diet/age effects on muscle mass present (mixed model analyzes with post hoc Sidak, P<0.05). Abbreviations: BW=body weight, FI=food intake, SUM=sum of hind limb of absolute muscle mass, TA=tibialis anterior muscle, EDL=extensor digitorum longus muscle, SOL=soleus muscle, PLANT=plantaris muscle, GM=gastrocnemius muscle, Ctrl=control mice, Lowox=mice on diet low in antioxidants, AOX=antioxidant supplemented group, PROT=leucine-enriched whey protein substituted group, TOTAL=combination of AOX and PROT strategy.

#### Muscle strength, function and quality

**Figure 5:**
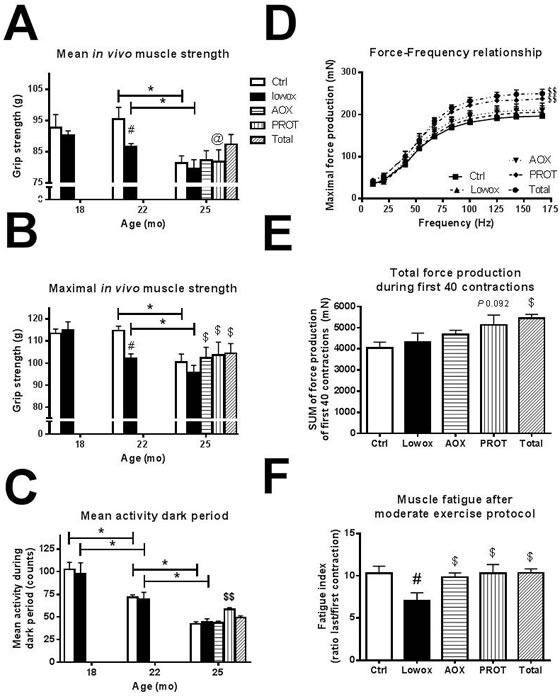
Muscle functionalities **A.** Absolute maximal forelimb strength values. At t0 and t4: ctrl *n* = 43 and lowox *n* = 42, at t7: ctrl *n* = 19, lowox *n* = 18, AOX *n* = 18, PROT *n* = 14 and TOTAL *n* = 17. **B.** mean daily physical activity during dark (active) period. At t0, t4 and t7: ctrl *n* = 10, lowox *n* = 13, AOX *n* = 10, PROT *n* = 6 and TOTAL *n* = 10. **C.** Force-frequency relationship at t7, **D.** Total force production during a moderate exercise protocol of the first 40 contractions at t7 and **E.** Muscle fatigue after a moderate exercise protocol at t7. For Figure 4**D.**-4**F.** Ctrl *n* = 16, lowox *n* = 13, AOX *n* = 15, PROT *n* = 10 and TOTAL *n* = 14. * Statistically significant age effect between 2 time points, # significant effect of lowox compared to control diet at corresponding time point, $ nutritional intervention effect compared to lowox-diet at t7 and $$ nutritional intervention effect compared to control diet at t7 (mixed model with *post hoc* Sidak, *P* < 0.05).

Figure 5A shows that both control and lowox-mice display a decline in maximal *in vivo* muscle strength from 22 to 25 months of age (*P <* 0.001). Lowox-mice already have significantly lower muscle strength at 22 compared to 18 months of age (*P* = 0.011) and compared to control mice of 22 months of age (*P <* 0.00001). Figure 5B shows the mean physical activity during the dark period for control and lowox-mice, and it clearly shows that activity decreases during ageing in both groups with no effect of the lowox-diet.

*Ex vivo* muscle function measurements of isolated EDL muscles at 22 (data not shown) and 25 months of age showed no significant difference in maximal force production between control and lowox-mice (Figure 5C) nor in force production during a moderate exercise protocol (Figure 5D). However, mice subjected to a diet low in antioxidants for 7 months showed significantly more muscle fatigue (i.e. a lower fatigue index) than control mice of the same age of 25 months (*P* = 0.003) (Figure 5E).

**Figure 6:**
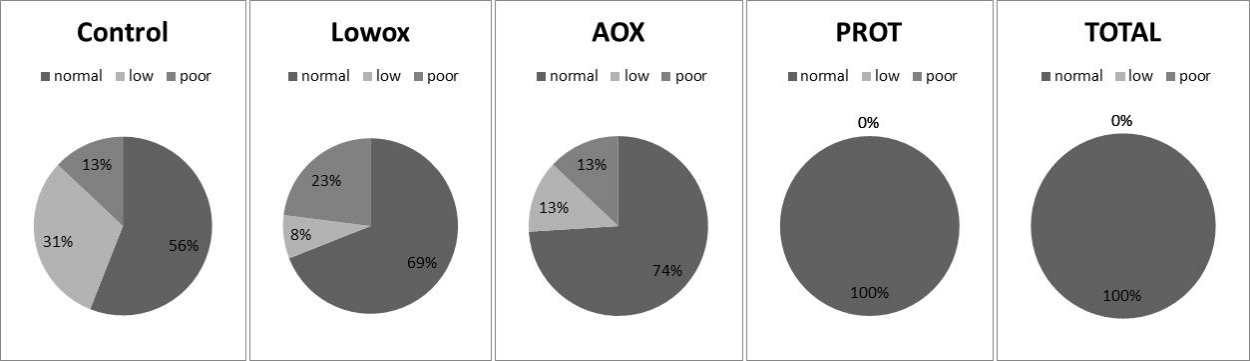
Muscle quality index Data was calculated as maximal *in vivo* grip strength divided by lean mass and was categorized as normal (> −1SD), low (−1SD to −2SD) and poor (< −2SD). SD was calculated from a young reference population and cut off values were: > 3.58 (= 1SD) > 2.62 (= 2SD). The next quality step was calculated as maximal *ex vivo* force production divided by EDL muscle mass and was categorized as well. Cut off values were: > 13.77 (= 1SD) > 10.53 (= 2SD). Statistical analyses were done using Fisher's exact test. There was a trend for improved muscle quality for the PROT group compared to the lowox group (*P* = 0.07) and the TOTAL group had significantly better muscle quality compared to the lowox group (*P* = 0.004).

Muscle quality, defined with an algorithm (Figure 2) showed no significant differences between the control and lowox-mice (Figure 6).

#### Mitochondrial dynamics

**Figure 7:**
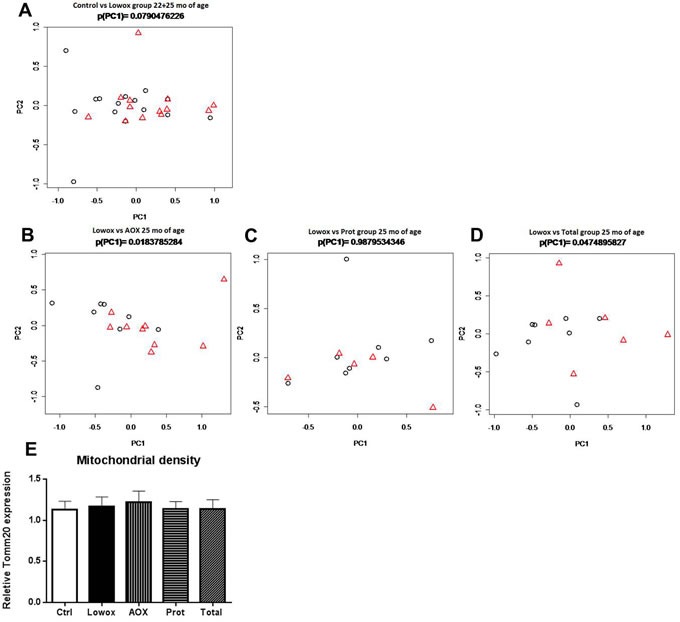
Mitochondrial dynamics PCA-based modulation of mitochondrial dynamics using all seven mitochondrial dynamics-related genes at age *t* = 25 months between respectively AOX **A.** PROT **B.** TOTAL **C.** compared to lowox reference group. Gene PCA-values of the mitochondrial dynamics gene set in EDL of Lowox-fed animals are represented by black circles, and experimental groups (AOX, PROT, TOTAL) are represented by red triangles **D.** PCA-based modulation of mitochondrial dynamics using all seven mitochondrial dynamics-related genes at age *t* = 22+25 months between control- and lowox-diet. Gene PCA-values of the mitochondrial dynamics gene set in EDL of Control-fed animals are represented by circles, and lowox are represented by triangles. **E.** gene expression of *Tomm20*, a marker of mitochondrial density.

Seven key genes involved in mitochondrial fusion and fission processes were analyzed: *Mfn1*, *Mfn2*, *Dnm1l*, *Fis1*, *Mff*, *Mief1*, and *Mief2*. To visualize the differences between the experimental groups, we applied a novel approach based on Principal Component Analysis (PCA). PCA showed that there was no significant difference between control and lowox-mice neither at 22 nor at 25 months of age and also no significant age effect. However, when both age groups were combined there was a trend (*P* = 0.079) that lowox-mice showed decreased levels of mitochondrial dynamics (Figure 7A). Mitochondrial density, i.e. gene expression levels of *Tomm20*, was not affected by a diet low in anti-oxidants at 25 months of age as shown in Figure 7E.

### Nutritional interventions in a mouse model with low dietary antioxidants

#### Oxidative status

As shown in Figure 3A and 3B, mice supplemented with antioxidants (AOX and TOTAL group) had significantly higher hepatic GSH levels and lower MDA levels compared to their lowox counterparts (*P* < 0.05). Plasma levels of vitamin A did not show any statistical differences between groups, but plasma levels of vitamin E clearly demonstrated higher levels of the antioxidant in the supplemented AOX and TOTAL diets. Also hepatic levels of both vitamins A and E clearly increased after supplementation of AOX and TOTAL compared to lowox-diet (*P* = 0.038, Table 3).

#### Body weight and composition

As Table 4 shows, no difference in body weight and food intake was observed between the 3 interventions compared to the lowox-mice. Lean mass was not significantly different in the AOX (*P =* 0.106) and PROT (*P* = 0.289) groups compared to lowox-mice. However, TOTAL-mice showed a significantly higher lean mass compared to lowox-mice (Figure 4A, *P* = 0.031). This effect on lean mass was not caused by an increase in muscle mass (Table 4) nor liver mass (data not shown). There was no difference in fat mass in the AOX, PROT and TOTAL group compared to lowox-mice (Figure 4B). The bone mineral density of the three nutritional interventions did not differ compared with the lowox group, and, similar to the lowox-mice, bone mineral density was higher compared to normal ageing mice at 25 months of age (Figure 4C, overall diet effect at 25 months of age *P* = 0.005).

#### Muscle strength, function and quality

As shown in Figure 5A, all interventions resulted in significantly higher maximal muscle strength at 25 months of age, compared with the lowox group (AOX: *P* = 0.007, PROT: *P =* 0.020 and TOTAL: *P* = 0.019, marked with $). The age-induced decline in physical activity during the dark period was significantly slowed down in the PROT group (*P* = 0.012), but not in the AOX or TOTAL group compared to lowox-mice at 25 months of age (Figure 5B). The physical activity level in the PROT group was higher than in normal ageing control mice at 25 months of age (*P* = 0.025).

During force-frequency relationship measurements of isolated EDL muscle, mice of the PROT and TOTAL group produced significantly higher maximal force than lowox-mice (*P* = 0.011 and *P* = 0.008 for PROT and TOTAL, respectively) and compared to control mice of the same age, as shown in Figure 5C. Additionally, during an exercise protocol, TOTAL-mice can produce significantly more force in the beginning of the exercise protocol than the other intervention groups and the lowox-mice, suggesting that these mice have more muscle power (Figure 5D). The PROT-mice showed a trend toward higher force production (*P* = 0.092 *vs*. lowox-mice). In contrast to the lowox-mice that show significant muscle fatigue at 25 months of age (*P* = 0.003), all three intervention groups were significantly less fatigued and had a fatigue index similar to the natural ageing control group (*P* = 0.012, Figure 5E).

Muscle quality was similar between lowox and AOX-mice at 25 months of age, but was significantly improved in the TOTAL group compared to the lowox-mice (*P* = 0.004). The PROT group showed a trend towards improved muscle quality (*P* = 0.07, Figure 6).

#### Mitochondrial dynamics

Evaluation on the whole process level clearly revealed that the mitochondrial dynamics gene set of 7 genes was significantly regulated in both the AOX group directly (*P* = 0.018) group (Figure 7B), as well as in the TOTAL group directly (*P =* 0.048, Figure 7D) group, compared to reference group (Lowox-mice). Gene expression levels of *Tomm20*, a marker for mitochondrial density, were similar between any of the groups (Figure 7E).

## DISCUSSION

This study clearly demonstrates the occurrence of lower maximal muscle strength and more muscle fatigue when mice were fed a diet low in antioxidants vitamin A, vitamin E, Selenium and Zinc (lowox-mice) compared to control mice of the same age. A (tendency towards) less efficient mitochondrial functioning may underlie these observations. In the present study, we showed that the impaired muscle quality linked to poor antioxidant intake during ageing can be improved by nutritional intervention in which dietary casein protein is substituted with a leucine-enriched whey protein source. This intervention also resulted in greater muscle force and maximal muscle grip strength and less muscle fatigue with advanced age. When lowox-mice were supplemented with elevated levels of the specific antioxidant mixture, the general oxidative status of the mice clearly improved, along with less muscle fatigue and improved maximal muscle grip strength. An improved mitochondrial function *via* more efficient mitochondrial dynamics could underlie these effects of antioxidant supplementation. A combination of the two different dietary strategies did not result in a synergistic effect, but showed the combined effects of both interventions with an improved muscle quality index, less muscle fatigue, improved muscle power and maximal grip strength. To our knowledge, a direct link between positive modulation of mitochondrial dynamics and dietary supplemented components with anti-oxidants properties has not been demonstrated so far. Here, we show that dietary antioxidant intervention regulates mitochondrial dynamics on the process of gene expression level, which is an important first indication that targeting mitochondrial dynamics contributes to improving muscle function. Moreover, the data provide evidence for improved clinically relevant parameters such as fatigue, grip strength, muscle power and muscle quality by adapting the dietary protein source without changing the amount of daily dietary protein intake.

The level of antioxidants in the diet low in antioxidants was 25% of the advised daily intake of vitamin A, E, Selenium and Zinc for rodents [47]. Compared with the high levels in AIN-93-M of these vitamins and minerals, which exceed the advised daily intake, the lowox-diet seems more representative of the human situation where deficiencies in micronutrients in older individuals are common [33-36]. The antioxidant levels in the lowox-diet were viable and achievable for the aged mice: their well-being was monitored daily with extra attention for weight loss, food intake and activity. Plasma and liver levels of vitamin A and E were not changed in the antioxidant deficient diet. This suggests that other mechanisms may compensate for the lower dietary intake or that other markers should be analyzed like other isoforms of the vitamins or enzymes involved in the corresponding metabolic pathways. After 7 months, the compensatory mechanism, however, seems compromised and oxidative stress increased further compared to the normal aging levels, as shown by an elevated level of the oxidative stress marker MDA in lowox-mice. This specific diet low in antioxidants did not significantly affect body weight compared to control mice, but it did affect body composition. The lowox-mice had less lean and fat mass after 7 months of the diet than control mice, but without any impact on skeletal muscle mass of the lower extremities. It is remarkable that all animals that received the lowox-diet during the first 4 months had higher BMD at 25 months of age, independent of the nutritional intervention thereafter and with no correlation to physical activity. The underlying mechanism and biological relevancy of this increased BMD are not clear.

Although muscle mass of the lower extremities did not differ between control and lowox-mice, the muscle strength of the upper extremities was significantly less in the lowox-mice, which was already apparent at 22 months of age. This may be similar to humans, who also display an earlier decline in muscle strength during ageing than muscle mass with a negative impact on activities of daily living [2, 40, 49]. The diet low in antioxidants did not affect physical activity in mice, indicating that the well-being of the lowox-mice was likely not impacted. The present study is unique in its experimental design to correlate long-term dietary antioxidant deficiency with increased muscle fatigue. A trend towards decreased mitochondrial dynamics in lowox-mice may contribute to this phenotype. Finally, muscle quality was not different between lowox and control mice at 25 months of age. In both groups, up to one third had a low or poor muscle quality, with a slightly higher number of mice with a poor quality in the lowox group.

The nutritional intervention had no influence on the amount of food intake. Antioxidants supplementation clearly improved the oxidative status of the mice as shown by reduced MDA levels and increased GSH levels, without any impact on body composition. These results are in line with previous studies in non-antioxidant deficient rodents where different combinations of antioxidants were supplemented [28, 50-52]. Maximal muscle grip strength and muscle fatigue after an exercise protocol were restored to age-control level. However, muscle quality index did not show any improvements. Mosoni *et al*. showed that the oxidative status was improved by antioxidant supplementation in aged rats, leading to a better oxidative status of other organs, like liver, spleen and heart [29]. Our data confirm this finding by showing an improved oxidative status of the liver. This improved oxidative liver status is also reflected in improved muscle function in a previous study in rats [52]. Because we used a combination of antioxidants, we cannot pinpoint the observed effect to one specific antioxidant. To further elucidate the mechanism by which antioxidants impact fitness, genes involved in mitochondrial dynamics were analyzed. PCA analyses demonstrated improved mitochondrial dynamics when supplemented with antioxidants, with no effect on mitochondrial density. Our results are in line with research of Lee *et al*. [53]; they state that mitochondrial homeostasis affects muscle fatigue by decreased Mfn1/2 expression. Another clinical study showed that in healthy, elderly men mitochondrial respiration is correlated to muscle fatigability [25]. Again another study demonstrated that mitochondrial biogenesis is involved in exercise response of healthy, elderly men [54], but no diet interventions were included. Research of Mohamed *et al*. in old mice showed that with increased skeletal muscle fatigue, SIRT-1 is down-regulated, leading to less mitochondrial control [55]. Therefore, it is plausible that mitochondria play a role in muscle fatigue and may be influenced by antioxidants.

Many different studies have used dietary whey protein in elderly individuals, rodents or men [16, 18, 25, 56-58]. Replacing casein protein with whey protein in the diet improved maximal muscle grip strength, muscle force production, resistance to fatigue, muscle power in the beginning of an exercise protocol and higher activity scores, without impacting muscle mass. Since only the protein source was replaced in the PROT and TOTAL groups (leucine-enriched whey instead of casein protein, resulting in higher dietary leucine levels of 16.8 *vs*. 13.0 g/kg) with no change in the protein quantity, this suggests that the protein source is important for muscle quality. Vianna *et al*. demonstrated that long-term leucine-enriched casein supplementation (29.3 g/kg) reduces fat mass gain without changing body protein status in ageing rats [59]. In this study, we did not find differences in body composition in the PROT group. This might be due to the difference in ageing process of mice *vs*. rats. Rats increase in body weight, mainly fat mass, throughout their lives [60], while body weight of mice stabilizes and subsequently decreases during ageing [61]. Norton *et al*. [15] demonstrated that dietary leucine content is a critical factor in stimulating postprandial muscle protein synthesis (MPS). However, we cannot exclude that MPS was frequently stimulated throughout the feeding period by higher levels of leucine in the diet. Even though muscle mass was not affected, a higher protein turnover could still have occurred, leading to improved (trend *P* = 0.07) muscle quality. Another hypothesis tested by Kanda *et al*. [57] was that dietary whey protein increases glycogen levels in muscle, leading to less muscle fatigue [62]. It has also been reported that depletion of muscle glycogen stores is associated with fatigue during endurance exercise [63, 64]. We could, however, not confirm this hypothesis as we observed no dietary effect on muscle glycogen levels (data not shown).

A combination of both nutritional strategies did not have synergistic effects. However, supplementing with only AOX or replacing the protein source by leucine-enriched whey protein in the PROT group did not show any effects on lean mass, while a combination of both significantly increased whole body lean mass. The AOX group showed less fatigue, likely through improved efficiency of mitochondrial dynamics; this was also applicable for the TOTAL group and therefore seems attributed to the antioxidant supplementation. The PROT group showed improved muscle strength and power, which was also demonstrated in the TOTAL group, with the addition that the muscle quality index was significantly increased in the TOTAL group (and only tended to improve in the PROT group). This suggests a benefit of the leucine-enriched whey protein source. These results support the hypothesis that both nutritional strategies contribute to an improved muscle quality, but likely act *via* different mechanisms.

In conclusion, the present findings show that a diet low in antioxidants leads to lower muscle strength, more muscle fatigue after an exercise challenge and to more oxidative stress without affecting skeletal muscle mass in mature mice. The negative impact of antioxidant deficiency could be prevented by supplementation of components with antioxidant properties. Supplementation of these components improves the oxidative status and also improves fatigue, which likely occurs *via* improved mitochondrial dynamics. Nutritional intervention with leucine-enriched whey protein can play a role in maintaining and improving muscle power and quality in aged mice with antioxidant deficiency. More importantly, we have demonstrated that supplementation of antioxidants in combination with a diet containing leucine-enriched whey protein is an effective and optimal strategy to improve general state of fitness and muscle quality in aged antioxidant deficient mice. These findings are also relevant for human aging, especially the elderly and frail or sarcopenic population that often suffers from antioxidant deficiencies, increased oxidative stress and loss of muscle quality. Therefore, clinical intervention studies in sarcopenic or frail older adults with antioxidant deficiencies are needed to confirm the observed effects on muscle health of antioxidants and leucine-enriched whey protein supplementation.

## MATERIALS AND METHODS

### Animals

Male C57/BL6J mice of 18 months of age were obtained from Janvier Labs (Saint Berthevin, France). Animals were individually housed in a climate-controlled room (12:12 dark-light cycle with a constant room temperature of 21±1°C). Mice were fed *ad libitum* a standard diet (AIN-93-M) and had free access to tap water. All diets were provided by Research Diets Services (Wijk bij Duurstede, the Netherlands). All experimental procedures were approved by the Animal Ethical Committee (DEC consult, Bilthoven, the Netherlands). After the acclimatization period, mice were randomized on body weight in two different groups. The mean body weight of the two different groups did not deviate more than 5% of the overall mean body weight. One group received AIN-93-M (control diet, ageing control group, *n* = 33) and the other group (*n* = 133) received a diet that was lower to 25% of the daily recommended intake of lab animals (see Table 1) [47] for vitamin A (retinol), vitamin E (α-tocopherol), selenium and zinc (from now on called lowox-diet). After 4 months, lowox-mice were randomized again on body weight and lean mass. Then the lowox-mice were randomly divided into the different nutritional intervention groups with no greater deviation than 5% from the overall lowox group mean for body weight and lean mass (*n* = 17 per group). For a period of 3 months, one group continued with the lowox-diet, the second group was supplemented with higher doses than the control group of vitamin A (3x), vitamin E (25x), selenium (13x) and zinc (6.5x) [29] (called AOX), in the third group the casein protein in the diet was replaced by whey protein with additional leucine (called PROT) and the final group received a combination of the diet of groups two and three (called TOTAL). Body weight and food intake were determined twice a week. Mean total food intake during the total experimental period of 7 months was calculated as well as daily food consumption. For a schematic overview of the experimental setup, see Figure 1. During the total duration of the experiment mice were carefully observed for symptoms of general malaise or signs of moribund conditions. Mice were sacrificed by block randomization at 22 or 25 months of age by cardiac puncture under total isoflurane anesthesia (isoflurane/N_2_O/O_2_), the skeletal muscles from the hind limb (*tibialis anterior*, *extensor digitorum longus* (*EDL)*, *soleus*, *plantaris* and *gastrocnemius* muscle) and the liver were dissected, weighted and stored at −80°C until further use.

### Daily activity

During the acclimatization period daily activity was measured for 1 week as normalization. Subsequently, daily activity was measured at the start of the experiment, after 4 months and at the end of the experiment on a subgroup of animals only due to practical limitations (*n* = 16/group). Physical activity was monitored continuously (24h) for 7 days using activity sensors (dual technology detector DUO 240, Visonic; adapted by R. Visser, NIN, Amsterdam, The Netherlands) that translated individual changes in the infrared pattern caused by movements of the animals into activity counts. Sensors were mounted above the home cages and were connected through input ports and an interface to a computer equipped with MED-PC^®^ IV software for data collection (MED associates, St Albans, VT, USA). Activity was expressed in counts per 30 min (both for the total 24h period, the dark period (active period) and the light period (inactive period)). Activity was calculated for each mouse separately. The daily activity measurement for one week was averaged, to reduce the day to day variability [65, 66].

### Body composition

Body composition, i.e. lean mass, fat mass and bone mineral density (BMD)/content (BMC) were measured by DEXA scan under general anesthesia using a PIXImus imager (GE Lunar, Madison, WI, USA).

### *In vivo* muscle strength

*In vivo* muscle strength was measured as forelimb grip-strength with a calibrated grip strength tester (Panlab, Cornella, Spain) by prompting the mouse to grip the trapeze bar with both forelimbs and pulling the mouse by the tail (proximal to the body) parallel to the orientation of the strain gauge and the trapeze bar. For mean grip strength analyses, a set included five repetitions. Data were calculated as follows: (a) absolute mean grip strength; (b) absolute maximum grip strength, defined as the maximum tension recorded over 5 repetitions.

### *Ex vivo* muscle function

Contractile characteristics of the *EDL* muscle were assessed *ex vivo*, as described previously [65, 67]. Briefly, muscles were allowed to stabilize in the organ bath (Hugo Sachs Elektronik, March-Hugstetten, Germany), for 30 min in Kreb's Heinselet buffer (mM: NaCl 118, KCl 4.75, MgSO_4_ 1.18, CaCl_2_ 2.5, KH_2_PO_4_ 1.17, NaHCO_3_ 24.9 and glucose 10) at 30°C and continuously perfused with 95% O_2_ and 5% CO_2_, after which optimal stimulation current and tension were determined. Subsequently, force-frequency characteristics (10-167 Hz, 250 ms) were determined. Isometric force signals of the force-frequency curve were analyzed for maximal and total force production [65]. After 5 min of rest and refreshing of the buffer, muscles were subjected to a moderate exercise protocol. Muscles were stimulated (143 Hz, 250 ms) 100 times. The maximal force production during the first 40 contractions of the exercise protocol was summarized and the ratio between the mean of the first 3 contractions and of the last 3 contractions was used as the fatigue index. Muscle power is defined as maximal force production divided by specific muscle mass (see Figure 2).

### Muscle quality index

Barbat-Artigas S *et al*. [2] propose an algorithm to define muscle quality, which we modified for mice as shown in Figure 2. The muscle quality index is based upon muscle mass, strength and power [2]. Therefore, adjusted muscle quality criteria were set for mice based on a 10 months old young reference group (*n* = 48, unpublished data). The reference population used to determine the muscle power/muscle mass ratio cut-points was composed of 48 C57/Bl6 mice of 10 months old. Low muscle quality was defined as a muscle power index value below 1 to 2 SDs of our reference population values; whereas poor muscle quality represented a muscle power index of 2 SDs or more (see Table 2).

### RT-qPCR method

Regulation of mitochondrial dynamics was investigated using Reverse Transcription quantitative real-time PCR (RT-qPCR) in EDL. Relative gene expression levels of the seven key genes that function in mitochondrial dynamics were evaluated, namely, mitofusin 1 and 2 (*Mfn1* and *Mfn2*), dynamin-1-like protein (*Dnm1l*), fission 1 *(Fis1*), mitochondrial fission factor (*Mff*), mitochondrial elongation factor 1 and 2 (*Mief1* and *Mief2)* [68]. Additionally, gene expression modulation of translocase of outer mitochondrial membrane 20 homolog (*Tomm20*) was studied to evaluate the effect on mitochondrial density. RNA was isolated with the RNeasy Fibrous Tissue Mini Kit (Qiagen) after high speed homogenization with the Tissuelyser II (Qiagen), both according to the manufacturer's protocol. Reverse transcription of RNA to cDNA was performed using the iScript™ cDNA Synthesis Kit (BioRad), according to the manufacturer's protocol, with an input of 750 ng RNA. RT-qPCRs were performed as we described earlier [69]. Relative expression levels of the genes of interest were normalized by the geometric mean of 3 reference genes according to the ΔΔCt method [70]. According to this method, ribosomal protein S15 (*Rps15*), hypoxanthine guanine phosphoribosyl transferase (*Hprt*), and ribosomal protein large P0 (*Rplp0*) were selected as reference genes. Primers that were used and their sequences are described in Supplementary Table 1.

### Biochemical measurements

Blood samples were taken *via* cardiac puncture and collected in tubes coated with heparin. Plasma was obtained by centrifugation at 1300 x *g* for 10 min at 4°C and stored at −80°C. Glucose levels were determined according to the GOD-PAP method [71]. Insulin was analyzed by ELISA (10-1247-01 Mercodia AB, Uppsala, Sweden). Plasma and liver samples for analyses of vitamin E and A were mixed with ethanol and centrifuged. The content of retinol and α-tocopherol was determined in the supernatant by HPLC, using UV-absorbance for detection of retinol and fluorometric properties for detection of α-tocopherol, by comparing with standard solutions [72].

Amino acid concentrations were analyzed in plasma and as free intracellular concentration in muscle by ultra-fast liquid chromatography (UFLC) [73]. For the latter, first the *tibialis anterior* muscle was cryo-desiccated [74], and muscle supernatants with 2% PCA were measured for non-protein bound intracellular free amino acid concentrations. Essential amino acids (EAA) were defined as the sum of Histidine, Threonine, Methionine, Tryptophan, Isoleucine, Leucine, Valine, Phenylalanine and Lysine.

### Malondialdehyde (MDA)

After cryo-desiccation of the total liver [74], 10 mg of the liver and the *plantaris* muscle was used to perform MDA analysis, a marker for whole-body lipid peroxidation. In short, thiobarbituric (TBA) reacts with MDA at pH3.5. TBA-MDA adduct is separated in a reversed-phase column and quantified by fluorescence detection (ex 515 nm; em 553 nm) [75].

### Liver glutathione (GSH)

To determine total glutathione (tGSH) content as a marker for whole-body oxidative stress, another 10 mg of cryo-desiccated liver was used. For tGSH analyses: 0.4 M perchloric acid was added. The samples were placed in an ultrasonic bath for 30 min, vortexed and centrifuged for 10 min at 13.000 rpm. The supernatant was diluted 100 times in phosphate-EDTA buffer (0.1 M, pH 7.5). Subsequently, to 40 μl sample and 20 μl DTNB (2.38 g/l in phosphate-EDTA buffer) reagent, 40 μl glutathione reductase (10 μl/ml in phosphate-EDTA buffer) and 100 μl NADPH (0.333 g/l in phosphate-EDTA buffer) were added, and the plate was read immediately. Measurements were performed every 21 s at 405 nm during 4 min. The maximal velocity was calculated over at least 7 measurements. Maximal velocity for the standards was plotted and a standard curve was constructed. Sample concentration was calculated as nmol/mg dry tissue.

### Statistical analyses

All data are expressed as means ± s.e.m. For randomization and all other experimental outcomes (except for gene expression data), statistical analyses were performed by use of a mixed model with *post hoc* SIDAK-Bonferronni testing, including mouse numbers, cohort, diet and age as covariates. To simplify the presentation of the results, after confirmation of no statistical differences between the different diet groups at the beginning of the experiment (prediet) and at the start of the intervention, groups were combined and are displayed as control *vs*. lowox group. *Ex vivo* skeletal muscle function data were analyzed by mixed model corrected for growth curve analyses with *post hoc* LSD. Muscle quality data were analyzed by Fisher's exact tests.

For statistical analyses on the individual gene expression level, data was checked for normality using D'Agostino-Pearson normality test, and if needed log-transformation was applied for normalization. Statistical differences between AOX and TOTAL *vs*. lowox groups of were analysed by using *t*-testing for normally distributed data, and for not-normally distributed data (even after log-transformation) by the non-parametric Mann-Whitney *U* test was applied.

To study effects on the process level we applied a novel approach based on Principal Component Analysis (PCA) [76]. In this analysis, a principal component is defined as a mathematically derived combination of genes and their expression characteristics that can be used to describe the process of interest. Here, we combined the gene expression data of all seven genes annotated to mitochondrial dynamics. A number of principal components that are mutually independent can be derived which in combination describe the process under study. Coordinates along the most discriminative principal component were calculated for each sample after PCA transformation. Deviation of respectively the AOX and TOTAL group to the reference group (lowox) was tested for significance by using a *t*-test.

Statistical analyses were performed using SPSS 19.0 (SPSS Benelux, Gorinchem, the Netherlands) and differences were considered significant at a *P*-value below 0.05.

## SUPPLEMENTARY MATERIAL TABLE


