# Supplementary material for: Improved muscle function and quality after diet intervention with leucine-enriched whey and antioxidants in antioxidant deficient aged mice
Source: Oncotarget. 2016 Feb 29;7(14):17338–55. doi: 10.18632/oncotarget.7800 (PMC4951216; doi:10.18632/oncotarget.7800)
Supplement: Supplementary file 1 [file oncotarget-07-17338-s001.pdf]

Improved muscle function and quality after diet intervention with leucine-enriched whey and antioxidants in antioxidant deficient aged mice

Supplementary Material

Supplementary Table 1. Primer sequences

Gene symbol	forward sequence (5' - 3')	Reverse sequence (5' - 3')
Mfn1	ATGCACAGAGGGTGCTGCTCG	TGGGCTGCATTATCCGGGGC
Mfn2	CGCCAGTTTGTGGAATACGC	CAGCCAGCTTTATTCCTGAGC
Mff	AAGTGGCTCTCACCCTAGCA	CTGCCCCACTCACCAAATGT
Fis1	GGCCATGAAGAAAGATGGACTG	CGATGGTGAGGATGGACAGG
Dnm1l	AGGGGGTAAGCCCTGAGCCAA	TTGCAGCAGTGACGGCGAGG
Mief2	GCTCGATTGGTGCTAGGTGT	TGTCGATGAGCCGCTTTACA
Mief1	GTGACCCGGAGAGAGGTACT	TGTACTAATCGTCACACTCCGC
Tomm20	GCTGGGCTTTCCAAGTTACC	AGCATCTGGAACACTGGTGG
Rps15	CGGAGATGGTGGGTAGCATGG	ACGGGTTTGTAGGTGATGGAGAAC
Hprt	TGACACTGGTAAAACAATGCAAACTTTG	GAGGTCCTTTTCACCAGCAAGCT
Rplp0	CAATAAGGTGCCAGCTGCTGCTCG	GAAGAAGGAGGTCTTCTCGGGTCCT
